# Hierarchical deep learning for predicting GO annotations by integrating protein knowledge

**DOI:** 10.1093/bioinformatics/btac536

**Published:** 2022-08-05

**Authors:** Gabriela A Merino, Rabie Saidi, Diego H Milone, Georgina Stegmayer, Maria J Martin

**Affiliations:** Bioengineering and Bioinformatics Research and Development Institute (IBB), FI-UNER, CONICET, Oro Verde 3100, Argentina; Research Institute for Signals, Systems and Computational Intelligence (sinc(i)), FICH-UNL, CONICET, Ciudad Universitaria UNL, Santa Fe 3000, Argentina; European Molecular Biology Laboratory, European Bioinformatics Institute (EMBL-EBI), Wellcome Genome Campus, Cambridge CB101SD, UK; European Molecular Biology Laboratory, European Bioinformatics Institute (EMBL-EBI), Wellcome Genome Campus, Cambridge CB101SD, UK; Research Institute for Signals, Systems and Computational Intelligence (sinc(i)), FICH-UNL, CONICET, Ciudad Universitaria UNL, Santa Fe 3000, Argentina; Research Institute for Signals, Systems and Computational Intelligence (sinc(i)), FICH-UNL, CONICET, Ciudad Universitaria UNL, Santa Fe 3000, Argentina; European Molecular Biology Laboratory, European Bioinformatics Institute (EMBL-EBI), Wellcome Genome Campus, Cambridge CB101SD, UK

## Abstract

**Motivation:**

Experimental testing and manual curation are the most precise ways for assigning Gene Ontology (GO) terms describing protein functions. However, they are expensive, time-consuming and cannot cope with the exponential growth of data generated by high-throughput sequencing methods. Hence, researchers need reliable computational systems to help fill the gap with automatic function prediction. The results of the last Critical Assessment of Function Annotation challenge revealed that GO-terms prediction remains a very challenging task. Recent developments on deep learning are significantly breaking out the frontiers leading to new knowledge in protein research thanks to the integration of data from multiple sources. However, deep models hitherto developed for functional prediction are mainly focused on sequence data and have not achieved breakthrough performances yet.

**Results:**

We propose DeeProtGO, a novel deep-learning model for predicting GO annotations by integrating protein knowledge. DeeProtGO was trained for solving 18 different prediction problems, defined by the three GO sub-ontologies, the type of proteins, and the taxonomic kingdom. Our experiments reported higher prediction quality when more protein knowledge is integrated. We also benchmarked DeeProtGO against state-of-the-art methods on public datasets, and showed it can effectively improve the prediction of GO annotations.

**Availability and implementation:**

DeeProtGO and a case of use are available at https://github.com/gamerino/DeeProtGO.

**Supplementary information:**

Supplementary data are available at *Bioinformatics* online.

## 1 Introduction

Proteins are involved in almost all biological processes (BPs) in the cell. Therefore, elucidating their functions, the processes they are involved in, as well as the cellular location where those processes are being done, is key for understanding how a biological system operates not only in normal conditions but also in a disease context (Li *et al.*, 2018). High-throughput sequencing efforts are driving increased coverage of the proteomes of thousands of organisms. However, providing high-quality information on the function of individual proteins requires experimental and manual techniques that are time-consuming and expensive. For instance, <600 thousands of the 215 millions of protein records in the UniProt Knowledgebase (UniProtKB, release January, 2021) have been reviewed by expert biocurators and deposited in the UniProtKB/Swiss-Prot repository (UniProt Consortium, 2019). Moreover, only about 0.1% of proteins in UniProtKB have at least one manually curated or experimental annotation. As the number of sequenced genomes rapidly grows, the overwhelming amount of newly discovered proteins can only be annotated initially by computational methods, which must provide a reasonable trade-off between precision and recall. Thus, automatic function prediction (AFP) tools become essential to reduce the gap between sequenced proteins and experimental annotations (Jiang *et al.*, 2016).

The most comprehensive and widely used database for protein functions annotations is the Gene Ontology (GO; http://geneontology.org). The GO knowledgebase is structured using a formal ontology involving classes of gene functions (GO terms) (The Gene Ontology Consortium, 2019). Each GO term represents a unique functional attribute and all terms are associated with each other in a directed acyclic graph (DAG) structure based on inheritance relationships. GO is organized in three DAGs, or sub-ontologies: molecular function (MF), BP and cellular component (CC). AFP methods deal with the computational assignment of GO terms to proteins of unknown or incomplete function from proteins whose function has already been manually curated and/or determined experimentally. Many approaches have been proposed for solving the AFP problem (Cruz *et al.*, 2017; Friedberg, 2006; Zhou *et al.*, 2019). These different strategies can be grouped into three categories: transfer based on sequence/homology, structure-based and systems biology-based (Cruz *et al.*, 2017). Sequence- and structure-based methods assume that proteins similar in sequence/structure have similar functionality, thus they search for sequence domains, structural features or multi-sequence alignments to infer functions. In this scope, sequence-based methods are more popular since it is experimentally more challenging to identify protein structures than sequences. Since proteins do not act individually, the third category of AFP methods is based on co-expression networks and protein–protein interactions (PPIs), which have shown to be good predictors for complex BPs (Cruz *et al.*, 2017; Rost *et al.*, 2003). Thus, integrating protein knowledge available through databases and literature could improve the AFP quality since they contain implicit and explicit descriptions of proteins and their functions (You *et al.*, 2019; Zhou *et al.*, 2019). Therefore, computational methods that accurately predict protein functions, considering not only sequence but also all related protein knowledge, and being applicable to proteins that have not been previously studied, and also to those whose annotations must be completed are needed.

Many new computational methods for AFP are published every year, which are mainly based on machine learning (ML) and methods for similarity search (Bonetta and Valentino, 2020). In order to provide a fair and equitative framework for their comparison, systematic benchmarking efforts have been developed by the community. The Critical Assessment of Function Annotation (CAFA) challenge tries to solve this problem by providing a real blind test and identifying the most effective methods for the AFP problem. The last challenge results [CAFA3 (Zhou *et al.*, 2019)] indicate that ML and sequence alignment remain the most used approaches for AFP in the three GO sub-ontologies. In addition, results revealed that top performing tools are mainly ensemble methods. For instance, GOLabeler (You *et al.*, 2018b) consistently outperformed the methods from all past CAFA challenges in the major categories. This method combines *k*-nearest neighbors using the popular Basic Local Alignment Search Tool (BLAST) (Altschul *et al.*, 1990) with logistic regression and a Naive computation of GO-term frequencies to solve the problem of learning to rank. For this, GOLabeler uses different features, such as: GO-term frequency, sequence alignment, amino acid trigram, domains, motifs and biophysical properties. Although method performances have shown an increase between CAFA2 and CAFA3, they are still a matter of improvement, even more for proteins without prior experimental annotation referred to as *no-knowledge* (NK) proteins. Indeed, the best tools achieved a CAFA F1 score that barely exceeded 0.4 in BP, and 0.6 in both MF and CC sub-ontologies for NK proteins (Zhou *et al.*, 2019). These numbers show that the problem of AFP is a long way from being solved and new approaches are still required (Makrodimitris *et al.*, 2020).

The emergence of deep-learning (DL) to model complex patterns of multi-level data has revealed its potential to address many challenges in different research fields. In particular, recent works have reported DL as a powerful tool for mining protein big data to obtain valuable knowledge (Liu *et al.*, 2021; Shi *et al.*, 2021). DL has also been proposed for solving the AFP problem in the last years (Kulmanov and Hoehndorf, 2020; Littmann *et al.*, 2021; Rifaioglu *et al.*, 2019; You *et al.*, 2018a). Furthermore, novel tools based on the cutting-edge DL architectures, such as transformers and graph neural networks, were presented very recently (Cao and Shen, 2021; You *et al.*, 2021). However, there are still limitations that need to be addressed. In this sense, hitherto developed DL methods have not been focused on integrating the heterogeneous available protein knowledge, but mainly designed for predicting GO terms using only protein sequences. For instance, DeepGOPlus (Kulmanov and Hoehndorf, 2020) uses the raw protein sequence as inputs of a deep convolutional neural network being this enough for improving the performance of state-of-the-art tools, such as DeepText2GO, based on text semantic representation (You *et al.*, 2018a). Similarly, DEEPred (Rifaioglu *et al.*, 2019), a stack of multi-task feed-forward networks, predicts GO terms from features calculated from the protein sequence. Meanwhile, goPredSim (Littmann *et al.*, 2021) is a method for annotation transfer based on similarity of protein-sequence embeddings (Embs) obtained from DL models. Furthermore, most of these models do not have full coverage of the ontology since they have been restricted to predict only those GO terms which have already been assigned to a minimum amount of proteins. It should be mentioned the importance of method comparisons on the same exact and standard test set. Usually, some reported scores are higher than those reached by CAFA3 winners but are based on their own datasets instead of standard data provided by CAFA community. Evenmore, sometimes the reported results refer to the whole CAFA3 benchmark set, masking the hardest challenge of NK proteins function prediction.

Here, we propose DeeProtGO, a DL model for predicting GO terms integrating protein data from multiple sources. To address the problem of the diversity in the type and amount of knowledge currently available for proteins, our approach considers different inputs ranging from only the sequence to incorporating co-occurrence of GO annotations, previously known GO annotations and sequence similarity. We show how the combination of more than one type of protein information could improve the prediction quality. Unlike other approaches, our method is easily adaptable for predicting terms from any of the GO sub-ontologies, without restrictions on the number of terms and providing high coverage. We evaluated our models using the CAFA3 challenge training and benchmark datasets, achieving scores that indicate DeeProtGO outperforms several CAFA3 top methods and state-of-the-art DL algorithms.

## 2 DeeProtGO model

### 2.1 Prediction tasks

According to the CAFA rules, the AFP challenge involves a timeline with three time-points that are considered to build the sets of proteins used as training and benchmark datasets. For CAFA3, $t_{-1}$ is when the challenge was released providing training and target proteins to the participants (September, 2016); *t*_0_, the deadline for participants submissions of the predictions for the target proteins (February, 2017); and *t*_1_ is when benchmark proteins were collected for assessment (November, 2017) (Zhou *et al.*, 2019). Thus, the CAFA3 benchmark is composed of those target proteins that have, at least, one new functional annotation added during the growth period between *t*_0_ and *t*_1_. This dataset involves two classes of proteins. On the one hand, the NK proteins are those that do not have experimental annotations in any of the GO sub-ontologies at *t*_0_, but have accumulated at least one GO term with an experimental evidence code during the growth period. On the other hand, the *limited-knowledge* (LK) proteins are those which already had one or more GO terms experimentally annotated in at least one of the three sub-ontologies at *t*_0_ (Jiang *et al.*, 2016).

**Fig. 1.**
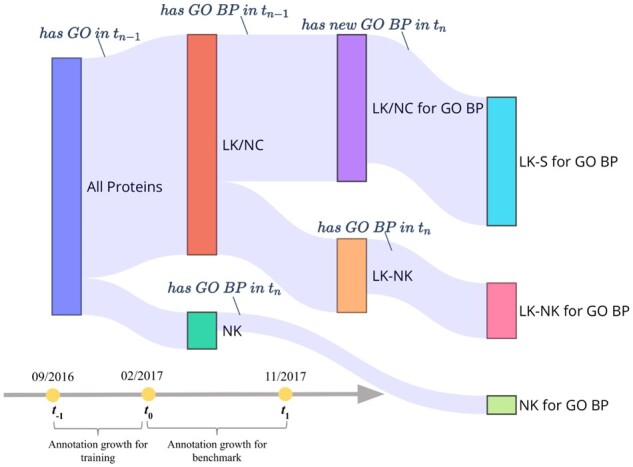
Schematic representation of protein classification for BP. Proteins are firstly grouped according to whether they had, or not, GORef $t_{n-1}$ (*n *=* *0 for training and *n *=* *1 for benchmark). The proteins having GO terms are defined as LK, and proteins whose annotation did not change between $t_{n-1}$ and *t_n_*, are named *No Change* (NC). Meanwhile, unannotated proteins are called *NK*. Focusing on the aimed sub-ontology, the LK/NC set is splitted into two groups: LK/NC for GO BP and LK-NK. From the first group, proteins that had BP terms at $t_{n-1}$ and that gained BP terms during the growth period (new annotations at *t_n_*) define the *LK-S* subset. Similarly, LK-NK proteins without BP terms at $t_{n-1}$ but gaining at least one BP annotation at *t_n_* are *LK-NK* for BP. Complementary, *NK* proteins are filtered to identify which of them were annotated with BP terms between $t_{n-1}$ and *t_n_*, referred as *NK* for GO BP

In order to fit models able to learn new annotations gained during a time gap, we defined a growth period between $t_{-1}$ and *t*_0_ specific for training. Thus, models presented here were trained using the protein knowledge available at $t_{-1}$ as input, to predict GO annotation at *t*_0_. Moreover, specific training and benchmark sets for each sub-ontology were generated, as it is shown in Figure 1. For this, proteins were classified based on their experimental annotations as follows:

- *LK-S*: Proteins *having at least one GO term in a particular sub-ontology* at the reference time ($t_{-1}$ for training, *t*_0_ for benchmark), and that have gained new annotations in this sub-ontology during the growth period.
- *LK-NK*: Proteins *without annotations in a particular GO sub-ontology* at the reference time, but that have been annotated in this sub-ontology during the growth period (i.e. the same as CAFA3 LK proteins).
- *NK*: CAFA3 NK proteins, i.e. *without GO annotations* at the reference time (GORef), but that have been annotated during the growth period.
- *Negatives*: Proteins *without annotations in a particular GO sub-ontology* at the reference time and that do not have gained annotations during the growth period.
- *No Change*: Proteins *do not change* their annotations during the growth period for a particular GO sub-ontology.

Taking into account this classification, three prediction problems for each GO sub-ontology were considered: (i) providing annotations for *NK* proteins, from which only the sequence and the taxon is the current information; (ii) predicting annotations for *LK-NK* proteins, from which only the sequence and the annotations in other GO sub-ontologies are the current information; and (iii) adding annotations for *LK-S* proteins, from which the sequence, the annotations in the other GO sub-ontologies, and the GO terms at the reference time in the sub-ontology to predict are the available information. In addition, in order to reduce the complexity of models, prokaryotic and eukaryotic proteins were modeled separately. Therefore, for each of the three prediction problems, we have developed two separate models (prokaryotic and eukaryotic) for each of the three GO sub-ontologies: BP, CC and MF. That makes a total of 18 AFP models.

### 2.2 Protein-knowledge representation

Protein information contained in amino acid sequences, organism taxa, InterPro annotations and GO annotations, were used here as a source of knowledge for AFP (Table 1). For *NK* models, sequence information was represented by means of two strategies. On the one hand, the SeqVec model (Heinzinger *et al.*, 2019) was used for obtaining sequence Emb of length 1024. On the other hand, sequence similarity between proteins of interest and the set of annotated proteins for each GO sub-ontology, was computed as the complement of the pairwise sequence edit distance (PSD) (Levenshtein, 1966; Raad *et al.*, 2020). In addition, organism taxa and InterPro annotations were represented using one-hot-encoding vectors. For all prediction tasks, GO annotations gained during the growth period, i.e. the prediction targets, were represented by using one-hot-encoding vectors.

**Table 1.** Types of input data used for training DeeProtGO for the three types of proteins, *NK*, *LK-NK* and *LK-S*

	PSD	Emb	Taxon	InterPro	GORef	GOCo1	GOCo2
*NK*	*✓*	*✓*	*✓*	*✓*	—	—	—
*LK-NK*	*✓*	*✓*	—	—	—	*✓*	*✓*
*LK-S*	*✓*	*✓*	—	—	*✓*	*✓*	*✓*

For *LK-NK* and *LK-S* problems, in addition to PSD and Emb sequence information, the GO knowledge relating each annotated protein with the other ones was represented by means of normalized co-occurrence vectors. For example, when predicting protein annotations for BP, there are two possible vectors of co-occurrence (GOCo1 and GOCo2) with respect to other proteins in MF and CC. These vectors indicate the number of terms in common between proteins at the reference time. In addition, the GORef of *LK-S* proteins were represented by a one-hot-encoding vector.

### 2.3 Architecture and training

**Fig. 2.**
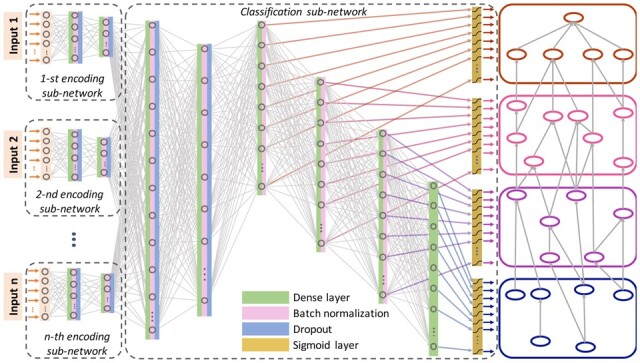
DeeProtGO schematic representation. Several possible types of inputs are shown at the left, with their corresponding encoding sub-network. The middle part shows the classification model architecture, including the hierarchical layer architecture to model the outputs. The hierarchical structure of outputs are depicted at the right, indicated with different colors

DeeProtGO is based on a feed-forward deep neural network that predicts the set of terms of a GO sub-ontology, by integrating several information sources with features built from the sequence and functional annotations of a protein (Fig. 2). Depending on the specific prediction task, *NK*, *LK-NK*, or *LK-S*, different numbers and types of inputs are considered. Thus, for providing the ability to learn specific features from each input, the model has several encoding sub-networks, one for each data type. Each of these sub-networks receives its corresponding input data and encodes them into a learned feature space. Each encoding sub-network has two fully connected layers with exponential linear units (ELU) as activation functions (Clevert *et al.*, 2015), batch normalization and dropout for model regularization. The output here is the set of learned features for the particular protein-knowledge input. These features are then concatenated into a single vector used as input for the classification sub-network.

The classification sub-network has six fully connected layers aimed to predict the full set of new GO terms for the protein under analysis. To take into account the ontology hierarchy, the last four layers are combined with sigmoid activation functions for modeling the output vector, being actually the deepest GO terms represented by the last output layer. That is, given the full set of output GO terms to be predicted, they are ranked according to the number of parents each term has. Then, the quartiles of this ranking are used to split the full set of GO terms into four hierarchically related vectors, used for training the last four layers. This configuration is shown in Figure 2 where the top-level predictions are colored in orange, the intermediate GO terms are indicated with pink and purple and the deepest GO terms, that come out from the last output layer of the model, are shown in blue. All the hidden layers of the classification sub-network also involve ELU activation functions, batch normalization and dropout.

DeeProtGO is trained in an end-to-end fashion using a cost function that is the sum of the loss of each layer representing the GO terms. To reduce the complexity of the hyperparameters optimization process, the optimizers and the loss functions were evaluated in first place only on a single model. The Adam optimizer (Kingma and Ba, 2014) is used and the loss at each layer is measured by using the binary cross-entropy classification loss,

(1) $$l_{\mathrm{BCE}}=\frac{1}{N}\sum_{i=1}^{N}\left[y_{i}\;\text{log}\;x_{i}+(1-y_{i})\;\text{log}(1-x_{i})\right],$$

where *y_i_* is the target label and *x_i_* is the predicted score for the *i-*th term in the output set of the *N* GO annotations.

## 3 Data and experimental setup

### 3.1 Data sources and datasets analysis

Data used for building and evaluating our models were obtained from different knowledge databases for the proteins of the CAFA3 challenge (https://www.biofunctionprediction.org/cafa/). Namely, a total of 66 841 proteins compose the training set, from which 58 717 belong to eukaryotic organisms and 8124 to prokaryotic species; a total of 3328 proteins compose the benchmark set, 2398 from 11 eukaryotic organisms and 345 belonging to 9 prokaryotic species. Sequence and organism data were downloaded from UniProtKB/SwissProt (version 2016_08). GO annotations at the three time-points defined in CAFA3 (Zhou *et al.*, 2019) were obtained from UniProt-GOA (version 158, 162 and 172 for $t_{-1}$, *t*_0_ and *t*_1_, respectively). Since manual curation or experimental validation are usually considered as highly reliable (Cruz *et al.*, 2017), only annotations with evidence codes EXP, IDA, IPI, IMP, IGI, IEP, TAS and IC were kept. These annotations were then propagated from the deepest terms to the top of the corresponding sub-ontology with GOATools (Klopfenstein *et al.*, 2018).

Since CAFA3 training set only provides proteins that have experimental annotations at $t_{-1}$ in order to obtain *NK* proteins for training, GO annotations at *t*_0_ and $t_{-1}$ for all UniProtKB/SwissProt proteins were compared. Thus, those proteins that did not have experimental annotations in any of the GO sub-ontologies at $t_{-1}$, but have accumulated at least one GO term with experimental evidence at *t*_0_, were also considered for training. In addition, proteins that had not changed their annotation in any sub-ontology were removed. As a result, the training set was composed of 49 875 eukaryotic and 7028 prokaryotic proteins with all their experimental GO annotations.

**Fig. 3.**
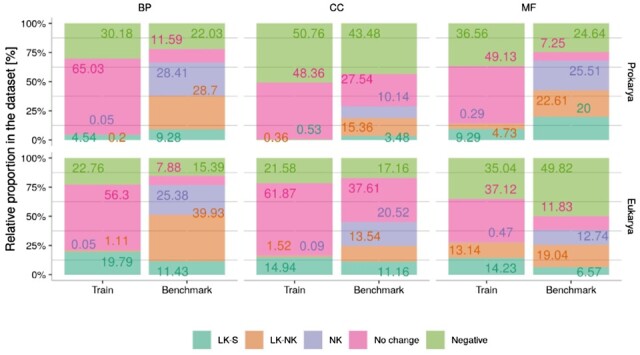
Detailed analysis of the CAFA3 challenge datasets for training and testing the DeeProtGO models. The training set involved 49 875 and 7028 proteins from eukaryotic and prokaryotic organisms, respectively; the amount of proteins in the CAFA3 benchmark dataset is 2983 (eukarya) and 345 (prokarya). The relative proportion of each type of protein (in percentage) in these datasets is shown along the *y*-axis

In order to define the training and benchmark datasets for each particular prediction task, the annotations for both train and benchmark CAFA3 proteins were analyzed. The Figure 3 shows, for each taxonomic kingdom and each GO sub-ontology (from left to right, BP, CC and MF, respectively), the percentage of *LK-S*, *LK-NK* and *NK* proteins, as well as of those *No Change* proteins, and those remaining as unannotated (*Negative* proteins). As it can be observed, splitting the prediction problem into the three sub-ontologies reveals the high percentage of *No Change* proteins (pink bars). Moreover, for all sub-ontologies in both prokarya and eukarya, the distribution of the different types of proteins highly differs between train and benchmark sets. This imbalance can affect not only the training process, but also the generalization capability of the learned model, when the distributions are very different between the two datasets.

### 3.2 Data augmentation and model tuning

Since DeeProtGO is aimed to predict new annotations acquired during a time gap, only those proteins gaining GO terms during the growth period represent *Positive* cases, while those that remain unannotated for a particular sub-ontology are those called *Negatives*. With this nomenclature, a protein could be for instance a *Positive* for the *NK* task in BP but a *Negative* for the *NK* task in CC. In addition, *No Change* proteins do not provide useful information for the model in terms of gaining new annotations. However, they can be strategically used for increasing the number of *Positive* proteins for some particular prediction cases, as a data augmentation strategy aimed to reduce the imbalance differences between train and benchmark sets. In the case of the *NK* model for a given sub-ontology, training proteins marked as *No Change* for that sub-ontology, and *Negatives* for the other two sub-ontologies, were used as *NK*. That is, these *No Change* proteins were considered unannotated at $t_{-1}$ while their annotations were supposed to be assigned during the growth period for model training $\left[t_{-1}\to t_{0}\right]$ for the sub-ontology of interest.

For each *LK-NK* model, *Positive* cases were augmented considering those proteins that are *No Change* not only for the target GO sub-ontology but also for, at least, one of the two other sub-ontologies. This criterion was established for ensuring the obtention of non-negative co-occurrences vectors that will be then used as inputs for these models. Thus, and like for *NK* augmented data, annotations of these *No Change* proteins used as *Positives* were ignored at $t_{-1}$ and assumed to be assigned during the growth period for model training $\left[t_{-1}\to t_{0}\right]$.

Although these strategies helped to increase the *Positive* cases, the percentage of *Negative* proteins was still higher in training than in benchmark for most prediction tasks. Thus, subsampling of *Negative* cases was also performed. The number of proteins finally kept as *Positives* and *Negatives* for each model is listed in Supplementary Table S1, as well as the number of GO terms to predict for each prediction task. It is worth to highlight these annotations cover all the GO terms available for the set of proteins of each prediction task, without any restriction related to term depth and/or representativity in the training sets.

DeeProtGO is implemented in PyTorch, in a user-friendly way to allow considering from one to six different inputs (code available at https://github.com/gamerino/DeeProtGO). The size of each input vector as well as of the output vector can be easily adapted for considering different data sources and modeling the different prediction tasks. Moreover, the implementation allows for optimizing hyperparameters, such as the number of neurons in hidden layers, dropout probability, batch size and learning rate, contributing to both the model scalability and the tuning process.

For each of the 18 prediction tasks, several alternatives using different sources of inputs were considered. Given the resulting size of the hyperparameter search space and since prokarya models are simpler than the eukaryotic ones, an extensive search for the number of neurons in hidden layers, dropout and batch size was performed for these models. Then, a smaller grid of these hyperparameters and only for the best combination of inputs were evaluated for eukarya models.

To standardize the setting of the units in the two hidden layers of the encoding sub-networks, proportions (ratios) with respect to the size of the input layer were used. Similar procedure was done for the first two hidden layers of the classification sub-network. For these hyperparameters, proportions ranging from 0.25 to 1.2 were evaluated. For each model and in combination with the proportions previously mentioned, the batch size, learning rate and dropout probability were also fine-tuned, considering values in the sets {8, 16, 32, 64, 128, 256, 512, 1024}, {0.001, 0.005, 0.01, 0.05} and {0.25, 0.5, 0.75}, respectively. All parameters were optimized for model performance on the test set of a cross-validation (CV) procedure considering 70%, 10% and 20% of data for training, validation and testing, respectively, within the time frame $\left[t_{-1}\to t_{0}\right]$. In addition, the number of epochs was selected by using early stopping monitoring the loss in the validation sets, with a patience of 10 epochs.

### 3.3 Performance measures

The performance of DeeProtGO was assessed by using the standard CAFA evaluation metrics. The *F*_max_, a protein-centric *F*-measure computed over the set of prediction thresholds, was used as the main performance indicator. For obtaining it, precision and recall for the *i*-th protein at the *t*-th threshold should be firstly obtained with $p_{i}(t)=(\sum_{f}I(f\in P_{i}(t)\wedge f\in T_{i}))/\sum_{f}I(f\in P_{i}(t))$ and $r_{i}(t)=(\sum_{f}I(f\in P_{i}(t)\wedge f\in T_{i}))/\sum_{f}I(f\in T_{i})$, where I(·) is the identity function returning 1 if the condition is true and 0 otherwise, *f* is a GO term, $P_{i}(t)$ is the set of predicted terms for the protein *i* at the threshold *t* and *T_i_* is the set of true annotations of protein *i*.

Average precision and recall are then obtained as

(2) $$\tilde{p}(t)=\frac{\sum_{i=1}^{m(t)}p_{i}(t)}{m(t)};\;\tilde{r}(t)=\frac{\sum_{i=1}^{n}r_{i}(t)}{n},$$

where *m*(*t*) is the number of proteins with at least one predicted GO term, and *n* is the total of proteins with true annotations. *F*_max_ is computed as

(3) $$F_{\max}=\underset{t}{\max}\left\{\frac{2\tilde{p}(t)\tilde{r}(t)}{\tilde{p}(t)+\tilde{r}(t)}\right\},$$

considering t∈[0,1] with a step size of 0.01 (Radivojac *et al.*, 2013).

## 4 Results and discussion

### 4.1 Hyperparameters analysis

DeeProtGO has been trained for solving the 18 tasks defined by the combination of the three GO sub-ontologies (BP, CC and MF), the three types of protein sets (*NK*, *LK-NK* and *LK-S*) and the two taxonomic groups (prokarya and eukarya). The average performance measures were calculated on the test partition within a 3-fold CV process in order to obtain an internal evaluation of hyperparameters for the proposed model. The optimization of hyperparameters involved training around a thousand models. The effect of the hyperparameters in the DeeProtGO performance was individually explored, evaluating the *F*_max_ in each case. A detailed analysis of the effect of batch size, dropout probability and the number of neurons in the hidden layers of the classification sub-network is presented in the section Hyperparameters analysis of the Supplementary Material.

One of the main hypotheses of DeeProtGO is that integrating heterogeneous protein knowledge can lead to a better and more effective annotation process. Supplementary Figures S1**A** and S2A reveal that this hypothesis is fulfilled for predicting BP and CC terms for *NK* proteins, where models integrating different types of data reached the highest *F*_max_ scores, in comparison with other single and unintegrated types of inputs. For both eukaryotic and prokaryotic proteins, when integrating Emb-Taxon DeeProtGO performs almost as well as when it uses PSD-Emb-Taxon as input. Thus, this indicates that the information useful for GO prediction represented by PSD may be contained in the Emb. Similarly, adding the input representing InterPro annotations did not have a great impact on the model performance, probably because domains information is already included in the sequence Emb representations. Thus, the increase in the number of parameters without adding new training examples leads to similar performance scores than those previously obtained without this new feature. Interestingly, for those proteins, the Emb data alone was enough for DeeProtGO to predict MF GO terms. For *LK-NK* model, combining Emb with the co-occurrence of GO annotations (GOCo1 and GOCo2) as model input led to the highest performances in both, eukaryotic and prokaryotic proteins (see Supplementary Figs S1B and S3A, respectively). Interestingly, combining PSD to these inputs led to poorer performance in the eukaryotic model for BP, the largest model in terms of the number of GO terms to be predicted (Supplementary Table S1). Since adding a fourth encoding sub-network implies significantly increasing the number of input dimensions and model parameters to be learned, it is possible that the number of training proteins was not enough for allowing DeeProtGO to reach the performance achieved when Emb, GOCo1 and GOCo2 were integrated. For *LK-S*, using annotations at the reference time (GORef) combined with PSD and Emb was enough for proteins from prokaryotic organisms (Supplementary Fig. S4A). Meanwhile, adding GOCo1 and GOCo2 improved the DeeProtGO performance for the eukaryotic case (Supplementary Fig. S1C). Thus, in summary, the more heterogeneous protein information DeeProtGO integrates, the more effective the prediction of GO terms is. In addition, one of DeeProtGO advantages is its flexibility for easily changing its inputs. For example, instead of the protein Embs from SeqVec, Embs obtained with newer methods, such as ProtT5 (Elnaggar *et al.*, 2021) can simply be used, which was in fact recently tested reaching a slight improvement in the DeeProtGO performance.

### 4.2 Performance on the training set

**Fig. 4.**
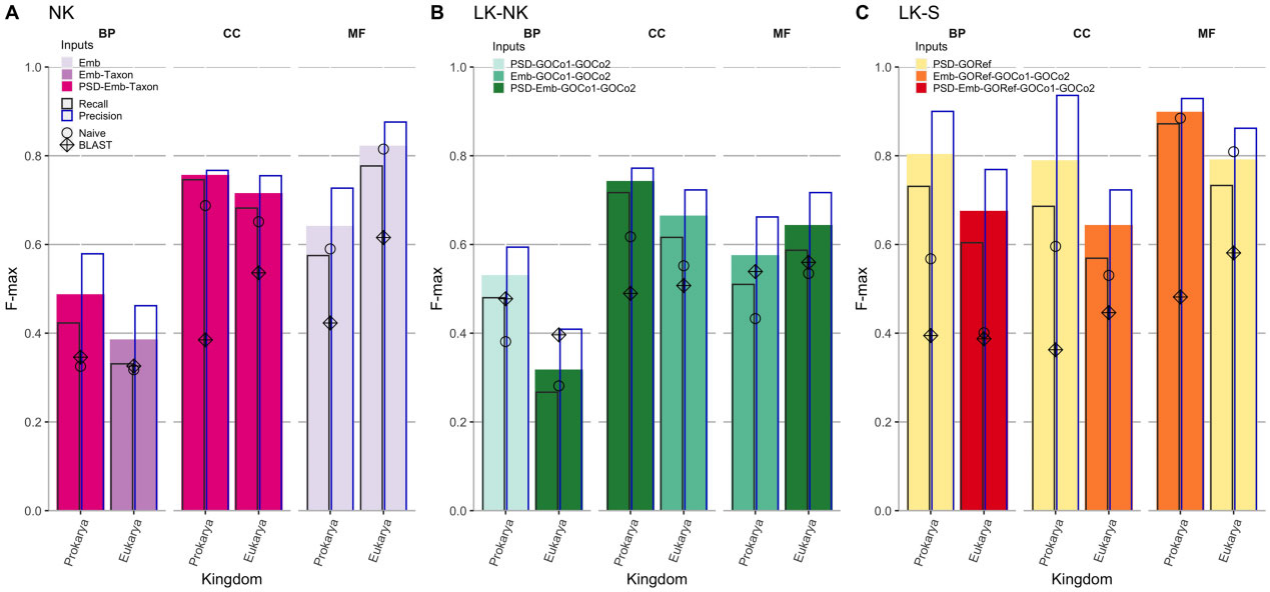
Performance of DeeProtGO and baseline methods during evaluation in a 3-fold CV scheme when predicting GO terms for (**A**) *NK*, (**B**) *LK-NK* and (**C**) *LK-S* proteins. Filled bars represent achieved *F*_max_ by DeeProtGO; empty bars represent recall (left) and precision (right) the recall and precision achieved at the *F*_max_, respectively. Colors filling the *F*_max_ bars indicate the input data combination used. Circles and diamonds indicate the *F*_max_ of Naive and BLAST, respectively

The performance measures of DeeProtGO on test partitions of the training dataset are depicted in Figure 4 and fully reported in Supplementary Table S2. The figure shows, for each prediction task (*NK*, *LK-NK* and *LK-S*) and each sub-ontology, the performance of DeeProtGO for each taxonomic kingdom. Filled bars represent the 3-folds average *F*_max_ reached by each model, with different colors indicating the best input integration. Within each filled bar, empty bars represent the corresponding recall (left) and precision (right), respectively. In addition, the *F*_max_ scores achieved by using baseline methods are also shown with circle (Naive) and diamond (BLAST) marks. These methods were implemented considering as predictor the subset of proteins with GOA that are in each training set defined in Section 2.1.

Results obtained with DeeProtGO for predicting annotations in *NK* proteins showed a pattern consistent with the state-of-the-art, where the highest scores were achieved for CC and MF sub-ontologies (Fig. 4A). In the case of BP, the *F*_max_ were 0.486 and 0.386 for prokarya and eukarya models, respectively, with precisions higher than recall in both cases. For predicting CC terms, observed *F*_max_ were higher than 0.700 being, as in the BP case, higher for prokaryotic organisms. The *F*_max_ found for the MF prediction was up to 0.823 for eukaryotic proteins and almost 0.642 for the prokaryotic ones. Interestingly, for both BP and CC, models reaching the best performance received information from sequence and taxon as input whereas MF models only used sequence data represented with Embs. Furthermore, and for the three sub-ontologies, DeeProtGO outperformed baseline methods.

This same experiment was repeated in order to evaluate how the hierarchical structure of GO contributes to the model performance. A flattened version of DeeProtGO, i.e. having a single output layer, was trained and tested in these six *NK* problems. Comparing the obtained scores (listed in Supplementary Table S3) against those previously reported (first rows in Supplementary Table S2) revealed that in *NK* models for BP in prokarya, and MF in prokarya and eukarya the *F*_max_ scores dropped up to 9% mainly because precision was reduced up to 16%. In the other three cases, although the *F*_ma__*x*_ values were similar to those reached using DeeProtGO in its original version, precision dropped between 2% and 8%.

Observed *F*_max_ scores in *LK-NK* proteins for DeeProtGO in each sub-ontology (Fig. 4B) were closer to those previously described, being the best models those having input information about GO-terms co-occurrences. For these proteins, predicting BP terms for both, prokarya and eukarya, is the most complex task because it requires training models able to learn how to assign more than 3600 and 15 000 GO terms, respectively. Indeed, for the eukarya prediction, the best model (*F*_max_=0.328) used input information from PSD, Emb and GO co-occurrences, altogether. Whereas, the *F*_max_ score of DeeProtGO for the prokarya prediction was almost below 0.541, but reaching a precision score up to 0.600. Similarly to *NK* models, the prediction scores for CC terms were higher for prokaryotic proteins than for the eukaryotic ones. Particularly, the best performance of DeeProtGO for *LK-NK* proteins was achieved in predicting this sub-ontology (*F*_max_ up to 0.743 and 0.665 for prokarya and eukarya, respectively). In addition, higher scores were reached when predicting MF terms for eukaryotic proteins. In the three sub-ontologies, it was also observed that DeeProtGO achieved higher precision than recall for both prokarya and eukarya cases. Same as for *NK* proteins, DeeProtGO outperformed baseline methods for most models.

In all *LK-S* tasks, shown in Figure 4C, the best DeeProtGO model integrates the annotation that LK-S proteins already have at the time of reference (GOref) with sequence information. Moreover, the *F*_max_ reached here is higher than those found in the *NK* and *LK-NK* problems, as it can be expected precisely due to this additional information. DeeProtGO performed very well for predicting BP terms of prokaryotic proteins, achieving an *F*_max_ up to 0.804 with a very high precision (0.900). Although the *F*_max_ was slightly lower than 0.700 of eukaryotic proteins, the corresponding precision was 0.769. Interestingly, the scores reached for predicting CC and BP terms for both eukaryotic and prokaryotic proteins were very similar, even revealing an extremely high precision up to 0.936. The best performance of DeeProtGO was reached when predicting MF terms, being the *F*_max_ almost 0.900 and 0.800 for prokarya and eukarya, respectively. Interestingly, for these groups of proteins, the Naive method outperformed BLAST in the three sub-ontologies although both reached lower scores than DeeProtGO. The high scores for the Naive method are due to the fact that this 3-fold CV setup is within training data, thus the train and test partitions have very similar distributions of GO annotation.

Our results confirmed that when the prediction problem is very complex, i.e. with less available information and, at the same time, a high number of GO terms to predict, the data integration process proposed by DeeProtGO is more effective and has a high impact on performance. In addition, consistently throughout the three sub-ontologies, it can be stated that DeeProtGO always exhibited a higher precision than recall, indicating its ability to assign true GO terms with fewer false positives. This is particularly important in the case of those proteins that have not been previously annotated, for helping the discovery of truly new knowledge.

### 4.3 DeeProtGO performance on CAFA3 benchmark data

The DeeProtGO model was also evaluated on the CAFA3 benchmark obtaining the results detailed in Table 2. The table shows, for each sub-ontology, and within it for each type of protein prediction problem and kingdom, the precision, recall and *F*_max_ obtained. Comparing DeeProtGO performance on benchmark with the achieved for the test partition in the 3-fold CV, the smallest drops were found in BP, the most challenging sub-ontology. Analyzing BP predictions revealed that the *F*_max_ ranged between 0.308 and 0.454. For *NK* proteins, the achieved *F*_max_ decreased in 35% and 10% in comparison with the scores observed for prokarya and eukarya, respectively, in the test set of the 3-fold CV experiments (shown in Fig. 4A). These results indicate that DeeProtGO is still good for predicting annotations for *NK*, mainly for eukaryotic proteins. Computing a weighted-average by the amount of proteins in each subset (according to the taxonomic kingdom) led to an overall *F*_max_ of 0.344 for *NK* proteins. Interestingly, the performance score observed for the eukarya *LK-NK* subset was higher in benchmark (*F*_max_=0.454) than in the CV test partition (*F*_max_=0.328, see Fig. 4B). A possible explanation for this result is that DeeProtGO for *LK-NK* has been trained using the proposed augmented data, which could have had a distribution of GO terms more similar to that of real *LK-NK* in the benchmark dataset. Moreover, note that given the very low number of true *LK-NK* proteins for BP (16 for prokarya and 652 for eukarya), training a model without data augmentation would have been practically impossible. Therefore, DeeProtGO was quite good at predicting the real subset of these annotations in the benchmark proteins. Interestingly, the largest drop of DeeProtGO performance was observed for *LK-S* models, where data augmentation was not performed. Thus, this result also supports our proposal of using data augmentation strategy to reduce the differences in the distribution of GO terms between the CAFA3 training and benchmark datasets. It is worth highlighting that the DeeProtGO precision for BP GO-terms prediction was always greater than the recall, as it was observed during model evaluation in the test set of the 3-CV.

**Table 2.** Performance of DeeProtGO models in the CAFA3 benchmark dataset

GO sub-ontology	Model	Kingdom	Recall	Precision	*F*_max_
BP	*NK*	Prokarya	0.270	0.383	0.317
		Eukarya	0.322	0.378	0.348
	*LK-NK*	Prokarya	0.303	0.372	0.334
		Eukarya	0.390	0.543	0.454
	*LK-S*	Prokarya	0.213	0.362	0.327
		Eukarya	0.257	0.383	0.308
CC	*NK*	Prokarya	0.326	0.324	0.325
		Eukarya	0.609	0.553	0.580
	*LK-NK*	Prokarya	0.369	0.541	0.439
		Eukarya	0.561	0.619	0.588
	*LK-S*	Prokarya	0.736	0.412	0.529
		Eukarya	0.543	0.489	0.514
MF	*NK*	Prokarya	0.455	0.554	0.500
		Eukarya	0.480	0.649	0.552
	*LK-NK*	Prokarya	0.384	0.585	0.463
		Eukarya	0.473	0.584	0.523
	*LK-S*	Prokarya	0.402	0.734	0.520
		Eukarya	0.456	0.649	0.536

In the case of CC predictions for eukarya, involving the largest amount of *NK* proteins, DeeProtGO performed very well, exhibiting an *F*_max_ closer to 0.600. Although the model performance for prokaryotic proteins (only 156 in the benchmark dataset) was 0.325, the weighted overall *F*_max_ for the full set of *NK* proteins was 0.546. Since true *NK* proteins for CC were not found during the training set construction for prokarya, DeeProtGO was purely trained with augmented data for this task. Although this could lead to the annotation of more terms than expected for a growth period like the one used for the CAFA3 benchmark, without data augmentation this task cannot be learned with a supervised approach. Similarly to the observed in BP terms prediction, the *F*_max_ drop between model evaluation in testing and benchmark was lower for models of the biggest set of proteins (eukarya) than for the smallest one (prokarya) in the three prediction groups, *NK*, *LK-NK* and *LK-S*. This is due to the large imbalance existing in CAFA3 training data regarding these kingdoms: there are very few examples (around one order of magnitude less) of prokaryotic proteins than eukaryotic ones, requiring a data augmentation strategy to train a predictor for such cases.

The best performance of DeeProtGO was for predicting MF GO terms. As it was previously found for most cases in the other two sub-ontologies, the scores reached for eukaryotic proteins were higher than those found for prokaryotic models. Thus, revealing DeeProtGO performance was good for predicting MF GO terms for most benchmark proteins achieving *F*_max_ higher than 0.520. Interestingly, the best DeeProtGO performance was found for predicting annotations of the hardest problem, represented by *NK* proteins. Evenmore, the high score achieved for eukaryotic proteins led to an overall *F*_max_=0.545 for the full set of *NK* proteins in the CAFA3 benchmark.

### 4.4 Comparison with state-of-the-art methods

**Fig. 5.**
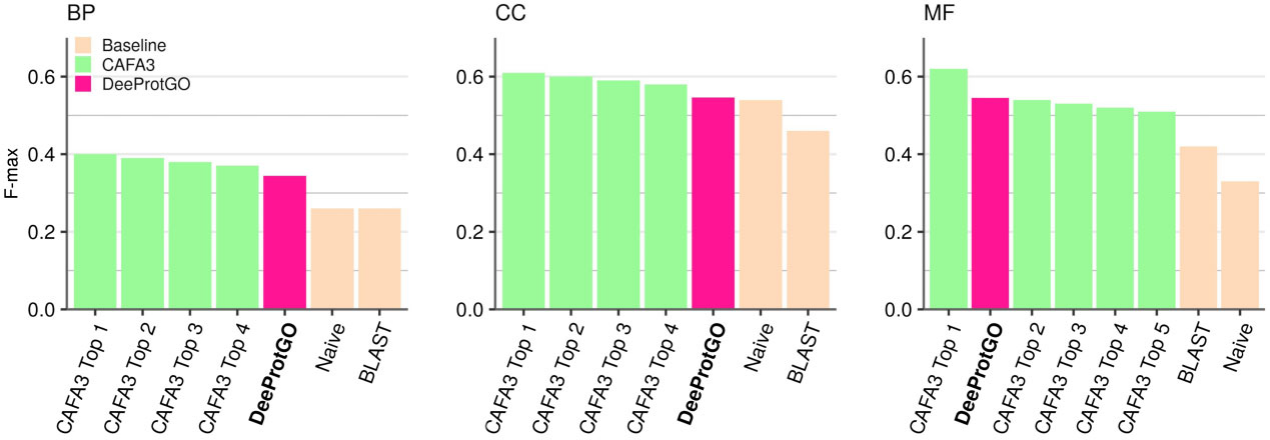
Performance comparison of DeeProtGO, top CAFA3 methods and baseline tools (Naive and BLAST predictors) when predicting GO annotations of NK proteins of the CAFA3 benchmark dataset

The overall *F*_max_ scores reached by DeeProtGO for *NK* proteins are shown in Figure 5. As it was previously mentioned, this *F*_max_ has been average weighted according to the number of proteins in each kingdom. In the same figure, for each sub-ontology, the performance is reported for baseline methods and the top models of the CAFA3 (Zhou *et al.*, 2019). Since several of the CAFA3 top models achieved the same scores, they were grouped under a single *F*_max_ value. Results reveal DeeProtGO has clearly outperformed the two baselines and it has performed in the top 5 CAFA3, achieving a score very similar to the one reached by the challenge competitors for BP. Predicting BP GO terms is still the most challenging problem in the AFP context, with the highest score barely exceeding 0.400 and with baseline predictions hovering around 0.300. Interestingly, only 4 of the top 10 methods in CAFA 3 achieved precision higher than recall at the *F*_max_, indicating their reliability to assign true GO terms with fewer false positives. This is especially important for predicting annotations of *NK* proteins. Meanwhile, DeeProtGO reported an *F*_max_ of 0.344, with precision up to 0.390 exceeding the recall (average value of 0.315).

For CC sub-ontology, similar results were obtained when comparing DeeProtGO (overall *F*_max_=0.546) and CAFA3 top methods. Moreover, the score achieved by DeeProtGO for eukaryotic proteins (*F*_max_=0.580) is very close to those shown in the Figure 5 of the CAFA3 report (Zhou *et al.*, 2019) (*F*_max_ of Top models for eukaryotic species between 0.600 and 0.630). Although this sub-ontology is the smallest one, the reason why one could think that predicting CC terms would be a very simple task, it has been described that CC is more complex than MF in terms of its graph structure (Peng *et al.*, 2018). This could explain, for instance, why both CAFA3 and DeeProtGO performances are similar and even closer to the score reached by the Naive approach.

The *F*_max_ reached by DeeProtGO in MF is 0.545, being it the second-best method when predicting MF terms of *NK* proteins in CAFA3 benchmark dataset. Interestingly, only two of the top CAFA3 models reported precision higher than 0.600 for these predictions. Meanwhile, DeeProtGO achieved a precision of 0.636, even higher than the corresponding recall (0.478). These results suggest that DeeProtGO outperformed most CAFA3 tools for AFP of MF terms in *NK* proteins, ensuring predictions with low rate of false positives. In addition, all the results presented here would have made DeeProtGO, one of the five best predictors for *NK* in BP and CC in CAFA3.

DeeProtGO was also compared against recent DL models published after the CAFA3 challenge. In order to perform a fair comparison, among several published methods only those reporting their performance separately for NK proteins of the CAFA3 were selected. Thus, the scores achieved by DeepGOPlus (Kulmanov and Hoehndorf, 2020), DEEPred (Rifaioglu *et al.*, 2019) and goPredSim (Littmann *et al.*, 2021) were extracted from their respective publications. For BP, the reported *F*_max_ are 0.390, 0.320 and 0.370, respectively. Thus, comparing them with the overall score for DeeProtGO (*F*_max_=0.344), our tool outperformed DEEPred, reaching an *F*_max_ closer to that obtained by goPredSim. A similar result was found for CC, being the reported *F*_max_ values 0.614 for DeepGOPlus, 0.340 for DEEPred, 0.570 for goPredSim and 0.546 for DeeProtGO. Meanwhile, for predicting MF terms, DeeProtGO (*F*_max_=0.545) outperformed both DEEPred (*F*_max_=0.490) and goPredSim (*F*_max_=0.500). It is worth noting that both DeepGOPlus and DEEPred models present an important restriction, differently from DeeProtGO, since they do not allow predicting the full set of GO terms of a particular sub-ontology. These models were developed for predicting only those terms annotated in more than 50 and 30 training proteins, respectively. Thus, aiming the models to focus only on those well-represented terms in the training dataset, perhaps makes them to miss very specific and precise GO terms describing a detailed protein functioning. Differently, it must be noticed that our proposal does not have such restrictions since DeeProtGO allows predicting all the terms in each sub-ontology that are present in the training dataset, which is a much harder problem.

Following the procedure used by DEEPred and DeepGOPlus, DeeProtGO was re-trained limiting the number of GO terms being learnt during training of *NK* models. Only those terms that are at least represented in 5% of proteins of the training dataset were considered. This restriction led to predict 80, 17 and 15 GO terms for BP, CC and MF, respectively, in prokaryotic proteins. While, the number of GO terms to predict for eukarya models was 331, 116 and 79, for BP, CC and MF, correspondingly. After exploring a small hyperparameter space using the training dataset, the reduced models were evaluated on the CAFA3 benchmark obtaining the performance scores listed in Table 3. Interestingly, this simplest version of DeeProtGO reached overall *F*_max_ higher than those previously reported in Table 2. Moreover, the improvement was larger for prokarya than for eukarya models, and for BP and CC sub-ontologies. For predicting BP terms, the new overall *F*_max_ is slightly lower than the score achieved by goPredSim but still higher than the reported by DEEPred. Evenmore, the *F*_max_ reached for eukaryotic proteins (*F*_max_=0.359) is similar to the values reported for the best CAFA3 models predicting all the proteins from this taxonomic kingdom (*F*_max_ from 0.360 to 0.400). The highest increase in the DeeProtGO performance when restricting the model output was observed when predicting CC terms for prokaryotic proteins. In fact, the new *F*_max_ for this *NK* subset is now in the range of the scores reported for the best models for prokaryotic organisms in CAFA3 (*F*_max_ from 0.380 to 0.460). The DeeProtGO performance observed for eukarya (*F*_max_=0.592) is also similar to those reported for CAFA3 top methods. Furthermore, the overall *F*_max_ resulted even higher than the ones reported by both DEEPred and goPredSim in this sub-ontology. For the MF sub-ontology, DeeProtGO still overperforms both DEEPred and goPredSim, achieving an *F*_max_ of 0.553 in eukaryotic proteins and an overall *F*_max_ of 0.547. Evenmore, DeeProtGO reached the score reported by DeepGOPlus in this sub-ontology (*F*_max_=0.557). Therefore, this experiment limiting the set of predicted terms, confirms DeeProtGO as one of the top predictors for BP, CC and MF terms of CAFA3 *NK* proteins outperforming both some of the top methods of CAFA3 and also some recent state-of-the-art methods.

**Table 3.** Performance of DeeProtGO in the *NK* CAFA3 benchmark dataset, predicting all GO terms when training with terms present in more than 5% of training proteins

GO sub-ontology	Kingdom	Recall	Precision	*F*_max_	Overall *F*_max_
BP	Prokarya	0.337	0.324	0.330	0.355
	Eukarya	0.321	0.407	0.359	—
CC	Prokarya	0.339	0.425	0.377	0.564
	Eukarya	0.559	0.628	0.592	—
MF	Prokarya	0.500	0.518	0.509	0.547
	Eukarya	0.476	0.660	0.553	—

*Note*: The overall *F*_max_ represents the score weighted-average by the number of proteins in each subset (156 for prokarya and 1024 for eukarya).

## 5 Conclusion

In this work, we have presented DeeProtGO, a DL model aimed to predict GO terms by integrating heterogeneous protein knowledge. Our model has been trained for solving 18 different AFP problems, defined by the GO sub-ontologies (BP; CCs; and MF), the type of proteins (*NK*; *LK-NK*; and *LK-Subset*, *LK-S*) and the taxonomic kingdom (Prokarya and Eukarya). Data from the third CAFA challenge (CAFA3) was exhaustively processed in order to define adequate training sets for each problem and data augmentation was used for increasing training cases in less represented groups. DeeProtGO has shown to improve its performance by successfully integrating heterogeneous protein information currently available. Moreover, and differently from other approaches, our proposal demonstrated to be easily adaptable for the 18 tasks, the different types of protein knowledge available, any number of terms from any of the GO sub-ontologies, without restrictions on the number of terms or annotated proteins and providing high coverage of protein functions.

Our experiments confirmed that the approach proposed here improves prediction results: the more protein information is integrated into DeeProtGO, the more effective the prediction of GO terms is. We demonstrated here the usefulness of DeeProtGO for predicting GO annotations for proteins. Evenmore, our model has achieved scores even higher than those reported by state-of-the-art methods for *NK* proteins. DeeProtGO has proved to be able to reliably predict likely annotations for proteins, with high precision, and without any restriction, enhancing the discovery of new functions. To improve the DeeProtGO performance, more experiments considering different protein knowledge, such as protein domains and PPI networks, and even implementing other DL architectures, for instance convolutional networks and transformers, will be carried out.

## Supplementary Material

btac536_Supplementary_Data
