# Supplementary material for: Hierarchical deep learning for predicting GO annotations by integrating protein knowledge
Source: Bioinformatics. 2022 Aug 5;38(19):4488–96. doi: 10.1093/bioinformatics/btac536 (PMC9524999; doi:10.1093/bioinformatics/btac536)
Supplement: btac536_Supplementary_Data [file btac536_supplementary_data.pdf]

Supplementary material

Hierarchical deep learning for predicting GO annotations by integrating protein knowledge

Gabriela A. Merino, Rabie Saidi, Diego H. Milone, Georgina Stegmayer, María J. Martin

Table S1: Number of proteins for models' training and evaluation in the CAFA3 benchmark dataset and number of GO terms to be predicted by each of the 18 DeeProtGO models.

Taxonomic kingdom	GO ontology	Model	GO terms to predict	Training proteins		Benchmark proteins	
				Positives	Negatives	Positives	Negatives
Eukarya	BP	*NK*	6,858	5,308	1,872	757	267
		LK-NK	15,300	25,238	3,862	1,191	192
		LK-S	6,710	11,636	7,366	341	235
	CC	*NK*	849	8,900	5,990	612	412
		LK-NK	1,930	12,692	12,424	404	100
		LK-S	1,119	8,788	36,383	333	1,122
	MF	*NK*	1,884	9,345	15,834	380	644
		LK-NK	5,016	24,318	20,565	568	842
		LK-S	1,704	8,369	15,075	196	353
Prokarya	BP	*NK*	1,690	1,050	621	98	58
		LK-NK	3,663	3,923	712	99	18
		LK-S	627	370	462	32	40
	CC	*NK*	173	1,234	2,397	35	121
		LK-NK	318	2,506	1,371	53	29
		LK-S	51	43	340	12	95
	MF	*NK*	817	1,194	922	88	68
		LK-NK	2,260	3,525	768	78	16
		LK-S	267	757	274	69	25

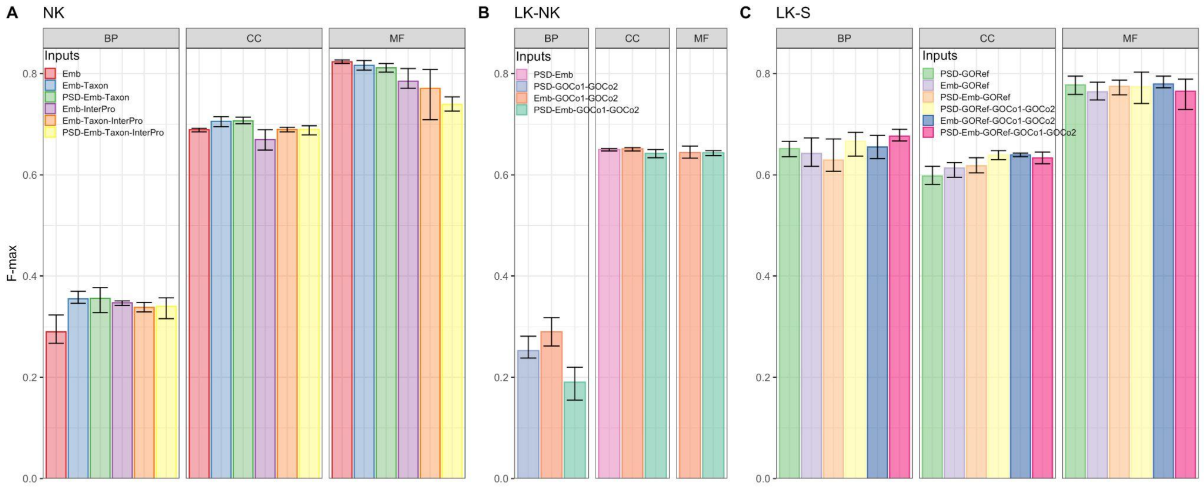

Figure S1: Each panel illustrates the effect of considering different inputs on the F_{max} achieved by DeeProtGO models when predicting annotations of eukaryotic proteins. Filled bars indicate the mean value and error bar, the minimum and maximum ones.The different model inputs are: similarity based on protein sequence edit distance (PSD), proteins sequence embeddings (Emb), organisms taxon, InterPro annotations, co-occurrence of GO annotations in the other two sub-ontologies (GOCo1 and GOCo2) and the GO annotations at the reference time (GORef). **A)** *NK* proteins, **B)** *LK-NK* proteins, **C)** *LK-S* proteins.

Hyperparameters analysis

Figure S2**B** shows the effect of **batch size** on DeeProtGO for each sub-ontology when predicting annotations for *NK* proteins from prokaryotic organisms for the two best input combinations. It can be seen here that, in most cases, similar patterns were found for each sub-ontologies, even when the model inputs were different. Particularly, 64 was the optimal value for the largest sub-ontology (BP), and 16 was the best batch size for the smallest one (CC). In the case of MF, the difference in model F_{max} achieved using batch sizes of 16 and 64 is around 0.01, suggesting any of those values can be used. Additionally, the patterns of F_{max} variation depending on batch size for each sub-ontology are very similar across models receiving different inputs, which was also observed for *LK-NK* (Figure S3**B**) and *LK-S* (Figure S4**B**) prokaryotic proteins. It should be noted that, in general, the incidence of batch size on F_{max} is around 3%, not having a large impact on the model performance.

The exploration of robustness to **dropout** probability for *NK* prokaryotic proteins is shown in Figure 1**C**. The results revealed that, in most cases, a dropout probability of 0.5 is the most adequate. Particularly, BP models using Emb-Taxon as input exhibited the highest robustness to this hyperparameter. Whereas, when PSD is also considered, increasing the dropout probability hyperparameter to 0.75 led to a model performance improvement. Interestingly, similar patterns of F_{max} variation found in BP models were observed for predicting GO terms in MF.

The analysis of variations in the **amount of neurons** in the two hidden layers of the classification model, expressed as a proportion of the number neurons in the corresponding input layer, is shown in Figure S1**D** for *NK* prokaryotic proteins. Results revealed variations lower than 0.05 in achieved F_{max} when using a fixed input, for most cases when predicting *NK* (Figure S1**D**) and *LK-NK* (Supplementary Material Figure S3**D**) prokaryotic proteins annotations, thus indicating DeeProtGO is robust to pHidden. Similar results were found for the *LK-S* prediction problem (Supplementary Material Figure S4**D**).

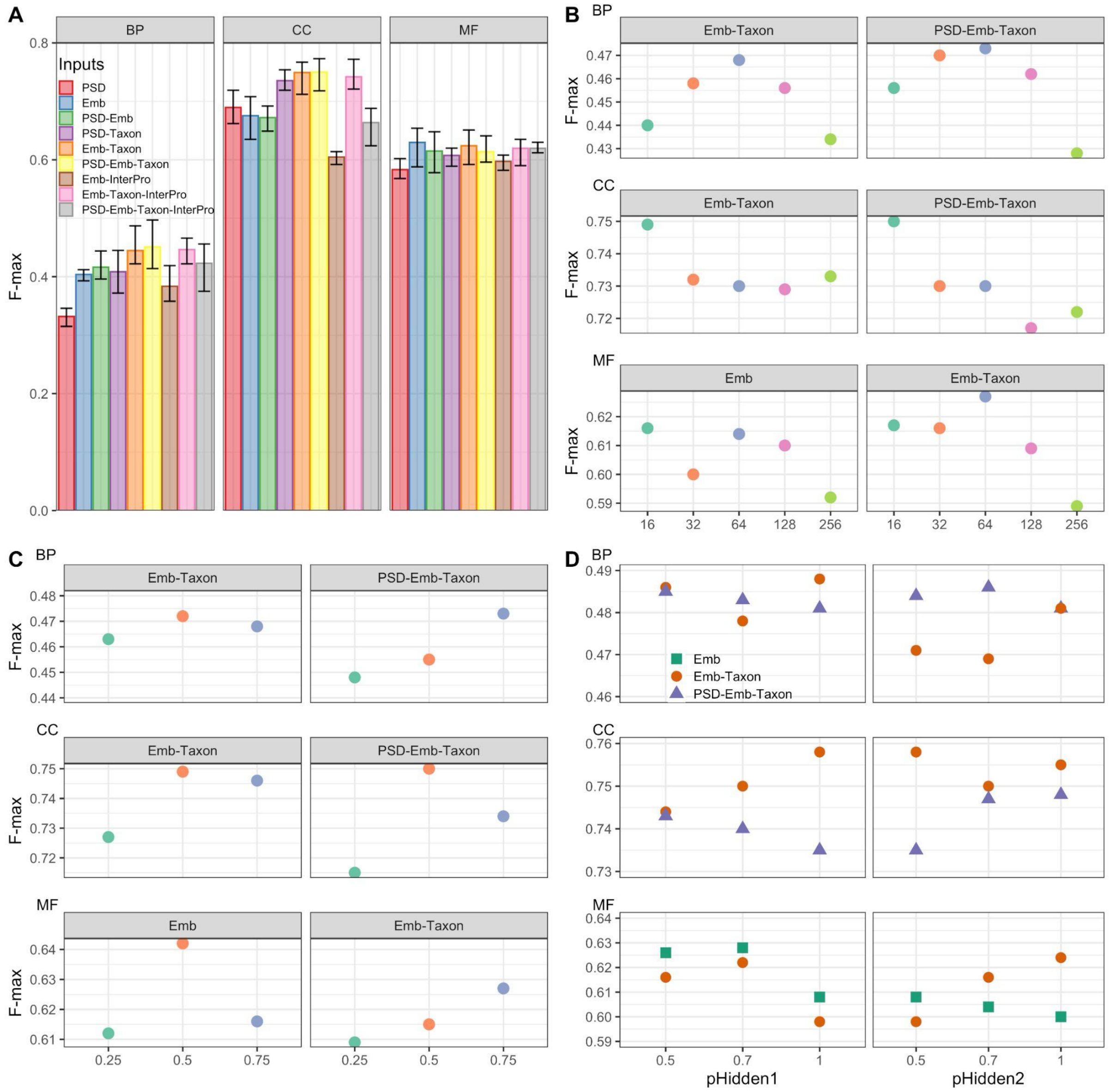

Figure S2: Exploration of model hyperparameters. Each panel illustrates the individual effect of hyperparameters on the F_{max} achieved by DeeProtGO models when predicting annotations of prokaryotic *NK* proteins. Assessed hyperparameters are: **A)** Model inputs combination: similarity based on protein sequence edit distance (PSD), proteins sequence embeddings (Emb), PSD-taxon, Emb-Taxon, PSD-Emb-Taxon, Emb-InterPro annotations, Emb-Taxon-InterPro, and PSD-Emb-Taxon-InterPro. Filled bars indicate the mean value and error bar, the minimum and maximum ones; **B)** Batch size; **C)** Dropout probability; **D)** Neurons in the two first hidden layers of the classification model, expressed as a proportion of the number neurons in the corresponding input layer.

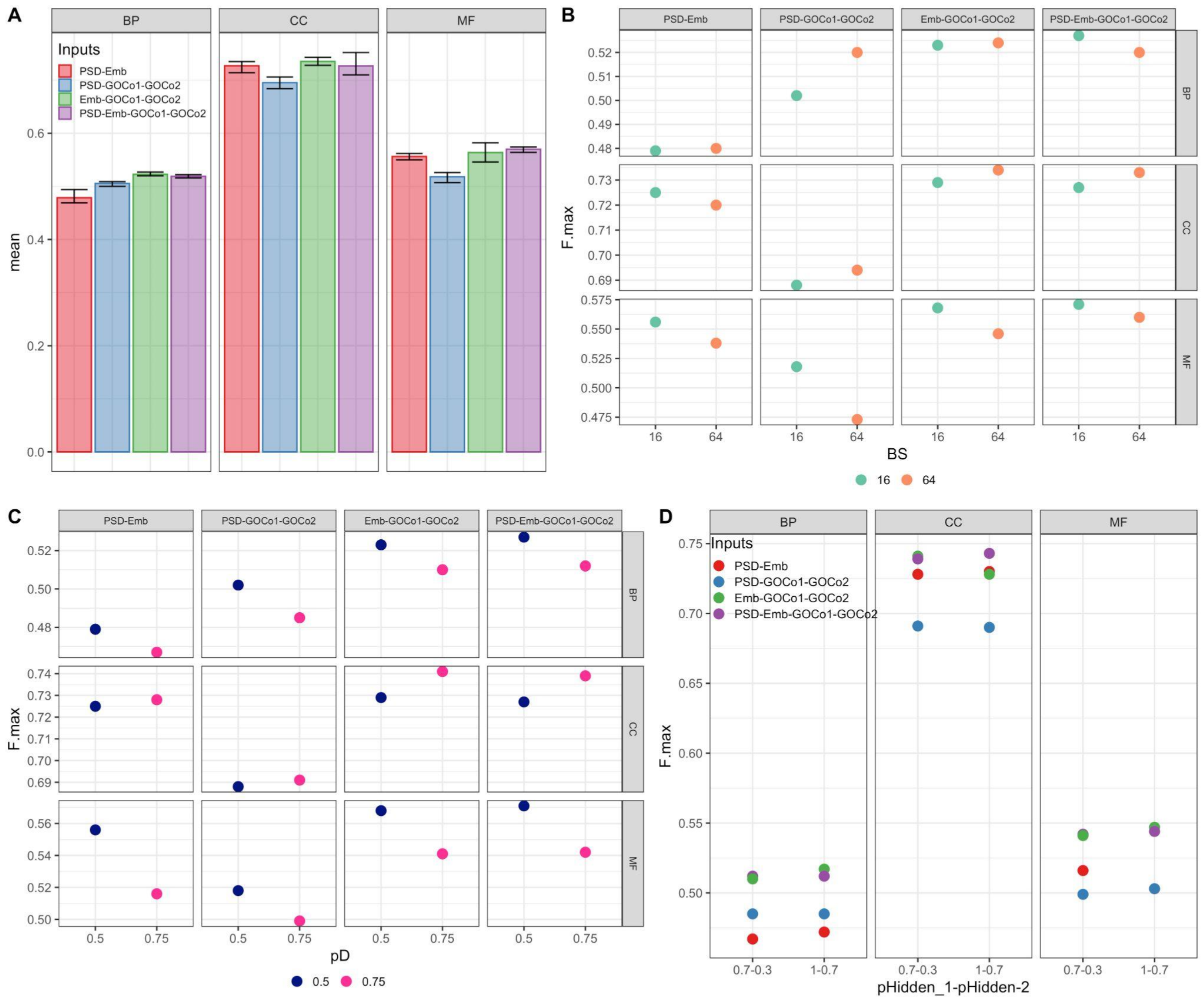

Figure S3: Exploration of model hyperparameters. Each panel illustrates the individual effect of hyperparameters on the F_{max} reached by DeeProtGO models when predicting annotations of prokaryotic *LK-NK* proteins. Assessed hyperparameters are: **A)** Model inputs combination, based on similarity based on the protein sequence edit distance (PSD), the proteins sequence embeddings (Emb), and the co-occurrence of GO annotations in the other two sub-ontologies (GOCo1 and GOCo2). Filled bars indicate the mean value and error bar, the minimum and maximum ones; **B)** Batch size; **C)** Dropout probability; **D)** Neurons in the two first hidden layers of the classification model, expressed as a proportion of the number neurons in the corresponding input layer.

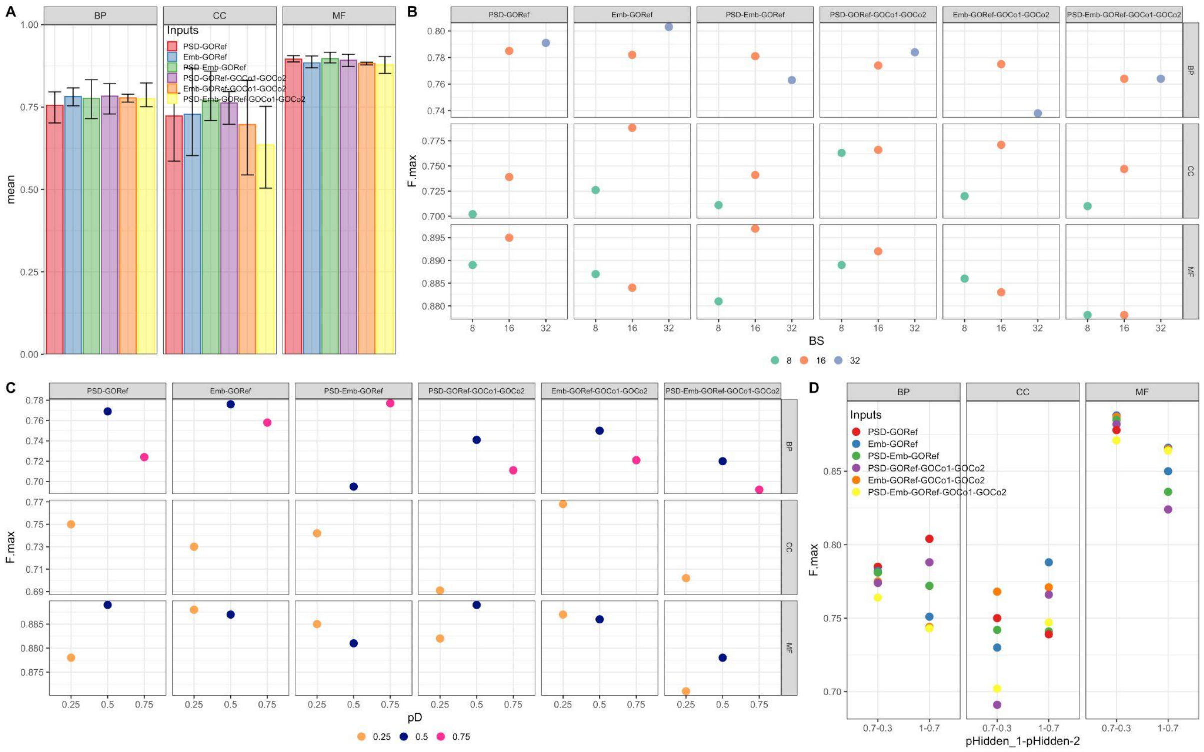

Figure S4: Exploration of model hyperparameters. Each panel illustrates the individual effect of hyperparameters on the F_{max} reached by DeeProtGO models when predicting annotations of prokaryotic *LK* proteins. Assessed hyperparameters are: **A)** Model inputs combination, based on similarity based on the protein sequence edit distance (PSD), the proteins sequence embeddings (Emb), the co-occurrence of GO annotations in the other two sub-ontologies (GOCo1 and GOCo2), and the GO annotations at the reference time (GORef). Filled bars indicate the mean value and error bar, the minimum and maximum ones; **B)** Batch size; **C)** Dropout probability; **D)** Neurons in the two first hidden layers of the classification model, expressed as a proportion of the number neurons in the corresponding input layer.

Table S2: Performance measures of the best DeeProtGO configurations and F_{max} scores reached by Naive and BLAST methods when predicting GO annotations during evaluation in a 3-fold cross-validation scheme.

				**DeeProtGO**			***Naive***	***BLAST***
Model	*GO sub-ontology*	*Kingdom*	*Input*	*Recall*	*Precision*	F_{max}	F_{max}	F_{max}
NK	BP	Prokarya	PSD-Emb-Taxon	0.423	0.579	**0.488**	0.325	0.346
		Eukarya	Emb-Taxon	0.331	0.462	**0.386**	0.318	0.326
	CC	Prokarya	PSD-Emb-Taxon	0.746	0.767	**0.757**	0.688	0.385
		Eukarya	PSD-Emb-Taxon	0.682	0.755	**0.716**	0.651	0.536
	MF	Prokarya	Emb	0.575	0.727	**0.642**	0.590	0.423
		Eukarya	Emb	0.777	0.876	**0.823**	0.815	0.616
LK-NK	BP	Prokarya	PSD-GOCo1-GOCo2	0.48	0.594	**0.531**	0.381	0.478
		Eukarya	PSD-Emb-GOCo1-GOCo2	0.267	0.409	0.318	0.281	**0.396**
	CC	Prokarya	PSD-Emb-GOCo1-GOCo2	0.717	0.772	**0.743**	0.617	0.490
		Eukarya	Emb-GOCo1-GOCo2	0.616	0.723	**0.665**	0.552	0.507
	MF	Prokarya	Emb-GOCo1-GOCo2	0.510	0.662	**0.576**	0.433	0.539
		Eukarya	PSD-Emb-GOCo1-GOCo2	0.587	0.717	**0.644**	0.535	0.560
LK-S	BP	Prokarya	PSD-GORef	0.731	0.900	**0.804**	0.568	0.395
		Eukarya	PSD-Emb-GORef-GOCo1-GOCo2	0.604	0.769	**0.676**	0.401	0.387
	CC	Prokarya	PSD-GORef	0.686	0.936	**0.790**	0.596	0.323
		Eukarya	Emb-GORef-GOCo1-GOCo2	0.569	0.723	**0.644**	0.530	0.446
	MF	Prokarya	Emb-GORef-GOCo1-GOCo2	0.872	0.929	**0.899**	0.885	0.482
		Eukarya	PSD-GORef	0.733	0.862	0.792	**0.809**	0.581

Table S3: Performance measures of *flattened* DeeProtGO when predicting GO annotations in the NK proteins set. The overall F_{max} represents the average score, weighted by the number of proteins in each subset (156 for prokarya, and 1024 for eukarya) for all the evaluations.

GO sub-ontology	*Kingdom*	*Flattened* DeeProtGO				*Hierarchical* DeeProtGO			
		Recall	*Precision*	F_{max}	*Overall* F_{max}	*Recall*	*Precision*	F_{max}	*Overall* F_{max}
BP	Prokarya	0.401	0.308	0.349	0.354	**0.423**	**0.579**	**0.488**	**0.399**
	Eukarya	**0.344**	0.367	0.355		0.331	**0.462**	**0.386**	
CC	Prokarya	0.367	0.388	0.378	0.547	**0.746**	**0.767**	**0.757**	**0.721**
	Eukarya	0.544	0.606	0.573		**0.682**	**0.755**	**0.716**	
MF	Prokarya	0.410	0.673	0.510	0.548	**0.575**	**0.727**	**0.642**	**0.799**
	Eukarya	0.470	0.676	0.554		**0.777**	**0.876**	**0.823**	
